# Cytokine activity in Parkinson’s disease

**DOI:** 10.1042/NS20220063

**Published:** 2023-12-04

**Authors:** Nicolas Dzamko

**Affiliations:** School of Medical Sciences, Faculty of Medicine and Health and the Charles Perkins Centre, University of Sydney, Camperdown, NSW, 2050, Australia

**Keywords:** cytokines, inflammation, interleukins, Parkinsons disease, tumour necrosis factors

## Abstract

The contribution of the immune system to the pathophysiology of neurodegenerative Parkinson’s disease (PD) is increasingly being recognised, with alterations in the innate and adaptive arms of the immune system underlying central and peripheral inflammation in PD. As chief modulators of the immune response, cytokines have been intensely studied in the field of PD both in terms of trying to understand their contribution to disease pathogenesis, and if they may comprise much needed therapeutic targets for a disease with no current modifying therapy. This review summarises current knowledge on key cytokines implicated in PD (TNFα, IL-6, IL-1β, IL-10, IL-4 and IL-1RA) that can modulate both pro-inflammatory and anti-inflammatory effects. Cytokine activity in PD is clearly a complicated process mediated by substantial cross-talk of signalling pathways and the need to balance pro- and anti-inflammatory effects. However, understanding cytokine activity may hold promise for unlocking new insight into PD and how it may be halted.

## Introduction

Parkinson’s disease (PD) is a debilitating neurodegenerative movement disorder with worldwide prevalence and impact. The progressive loss of dopamine producing neurons in the brain coupled with the spread and accumulation of pathogenic forms of α-synuclein protein results in a heterogenous array of symptoms that include motor dysfunction, depression, cognitive decline, sleep imbalance and gastrointestinal dysfunction [1]. Despite being recognised as a chronic disease for more than two hundred years, the exact cause(s) of PD remain unknown and current treatments are only symptomatic.

Along with the loss of dopaminergic neurons and accumulation of α-synuclein, the presence of neuroinflammation is a hallmark pathological feature of PD. Studies of post-mortem brain tissue from PD patients reveal a marked increase in activated microglia, the resident immune cells of the brain, particularly in the midbrain where dopaminergic neurons are localised [2]. Microglial activation is associated with a range of responses including the activation of complement, inflammasome and other immune signalling pathways that result in the secretion of cytokines and chemokines, as well as the induction of phagocytosis (reviewed in [3]). There is some debate as to whether microglial activation is detrimental or beneficial in the context of PD, and likely a fine balance is needed. Moreover, it is still unclear if microglial activation is a cause or consequence of PD, although at least some studies have shown microglial activation occurring early in the disease process using positron emission tomography (PET) to measure the microglial activation marker translocator protein of 18 kDa (TSPO) [4–6].

In addition to neuroinflammation there is also substantial evidence that peripheral inflammation plays a role in PD (Table 1). Indeed, the first clinical PD symptoms and potentially alpha-synuclein pathology are associated with the gut [7]. Altered peripheral immune responses and increased plasma/serum cytokines have also been observed in PD patients, including in early and prodromal cohorts [8,9]. A number of proteins encoded by PD risk such as leucine-rich repeat kinase 2 (LRRK2) and glucocerebrosidase (GCase) and highly expressed in peripheral monocytes, and in the case of the main familial PD risk gene *LRRK2*, there is overlapping genetic risk with inflammatory bowel disease and the susceptibility to bacterial infection [10,11]. Moreover, peripheral viral or bacterial challenges can induce PD like symptoms in preclinical models (eg [12–14].) and viral infections have been linked to a higher incidence of PD [15]. Peripheral immune T cells and monocytes can also infiltrate into PD brain and exacerbate neuroinflammatory responses in preclinical models [16,17]. Collectively, there is therefore compelling evidence that both peripheral and CNS immune responses contribute to the progression and possibly onset of PD.

**Table 1:** Cytokine changes in Parkinson’s disease

Study	Cohort	Cytokine changes in PD	Reference
Ortega-Vazquez et al.	*De novo* PD (27) and controls (22)	↓ IL17A in plasma	[128]
Chang et al.	PD (109) and controls (113)	↑ CCL5, ↑ TNFα in serum	[129]
Yacoubian et al.	*De novo* PD (56) and controls (62)	↑ CCL4 (14%) in plasma, ↑ CCL2 (27%), ↑ CCL17 (42%), ↑ CCL22 (36%) in CSF	[130]
Xiromerisiou et al.	PD (142) and controls (67)	↑ TNFα (40%) in serum	[131]
De Bartolo et al.	*De novo* PD (80) and controls (62)	↑ TNFα (68%) in saliva	[132]
Liu et al.	PD (22) and controls (22)	↑ IL10 (143%) in plasma	[133]
Roy et al.	PD (27) and controls (16)	↑ IL1β (na) in serum	[134]
Li et al.	PD (76 and 80) and controls (76 and 80) from 2 cohorts	↑ CXCL12 (36%) ↑ IL8 (114%) ↑ CX3CL1 (22%) in plasma	[135]
Kim et al.	PD (45) and controls (20)	↑ IL1β (66%) ↑ IL2 (40%) ↑ IL6 (137%) in serum	[136]
Diaz et al.	PD (26) and controls (14)	↑ IL6 (∼50%) in serum	[137]
Xu et al.	PD (32) and controls (30)	↑ IL1β (5%) ↑ IL33 (14%) in serum	[138]
Hu et al.	PD (139) and controls (30)	↑ IL1β (11%) ↓ TNFα(195%) in CSF	[139]
Chan et al.	PD (113) and controls (48)	↑ IL1β (na) ↑ TNFα (na) in plasma EVs	[140]
Nissen et al.	PD (106) and controls (16)	↑ IL8 (na) ↑ IL15 (na) in CSF	[141]
Borsche et al.	PD with PRKN/PINK mutations (15) and controls (90)	↑ IL6 (∼36%) in serum	[142]
Calvani et al.	PD (20) and controls (30)	↑ IL8 (212%) ↑ CCL4 (100%) ↓ IL9(27%) ↓ CCL3(500%) in serum	[143]
Chatterjee et al.	PD (27) and controls (15)	↑ IL1β (380%) in serum	[68]
Adams et al.	PD (39) and controls (39)	↑ IL1β (53%) ↑ IL17A (1270%) ↑ IL1α (52%) ↑ TNFα (94%) in plasma	[144]
Lin et al.	PD (120) and controls (120)	↑ TNFα (76%) ↑ IFNγ (17%) ↑ IL13 (326%) in plasma	[145]
Rathnayake et al.	PD (72) and controls (56)	↑ IL10 (na) ↑ IFNγ (na) in serum	[146]
Atashrazm et al.	PD (24) and controls (27)	↑ TNFα (14%) ↑ CCL5 (49%) ↑ GMCSF (66%) in plasma	[147]
Kim et al.	PD (58) and controls (20)	↑ IL1β (67%) in serum	[65]

A summary of cytokine changes in PD patient biofluids compared with controls from 2019 to present using a search of Pubmed for inflammation + cytokine + Parkinson’s disease. Only cytokines reported as being significantly different in PD are included. Only original research articles were included. Na = not available, i.e. raw values were not reported.

Although the definitive triggers of immune responses in PD remain to be elucidated, and may occur via a myriad of immune receptor pathways, invariably the response will involve the production and secretion of pro- and anti-inflammatory cytokines. Based on the literature and clinical studies shown in table 1, this review will highlight key cytokines that can modulate pro-inflammatory (TNFα, IL-6 and IL-1β) and anti-inflammatory (IL-10, IL-4 and IL-1RA) signalling pathways that may contribute to PD pathology.

## Key cytokines with pro-inflammatory actions that are implicated in Parkinson’s disease

**Figure 1:**
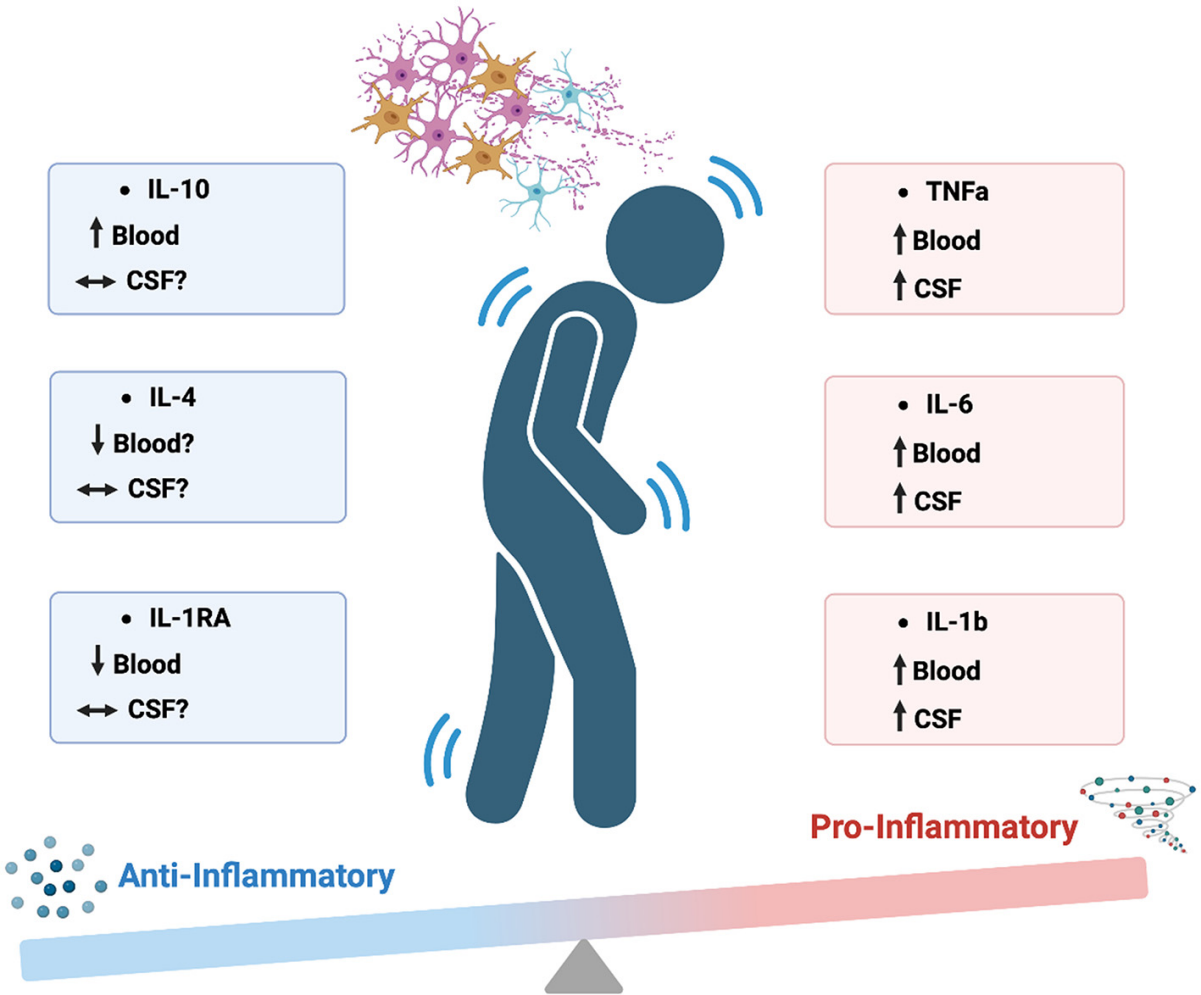
Cytokine imbalance in PD PD is associated with an imbalance in the levels of peripheral and central cytokines that mediate pro- and anti- inflammatory responses, resulting in a chronic low-grade pro-inflammatory phenotype. Pro-inflammatory cytokines can contribute to the degeneration of movement controlling dopamine neurons via the activation of microglia or by acting directly on neurons themselves. In turn, this contributes to the clinical symptoms of PD. Figure created with Biorender.com

Although many cytokines have pleotropic functions there are many cytokines considered pro-inflammatory that probably contribute to the chronic low-grade inflammatory milieu found in PD (Figure 1).

### Tumor necrosis factor α (TNFα)

Biological activity of TNFα is mediated via the receptors TNFR1 and TNFR2. TNFR1 is ubiquitously expressed and differs from TNFR2 by the presence of a death domain. Activation of TNFR1 by binding of soluble or transmembrane bound TNFα results in the death-domain dependent recruitment of TNFR1-associated death domain (TRADD) and receptor interacting protein kinase 1 (RIPK1), which form the basis of the TNFα signalling complex [18]. Further recruitment of TNF receptor-associated factor 2 (TRAF2) facilitates ubiquitination of the complex and recruitment and activation of transforming growth factor-β-activated kinase 1 (TAK1). Complete ubiquitination of the complex ultimately results in the activation of NFκB, p38 and JNK transcription factors, which collectively promote cytokine signalling and cell survival. Incomplete ubiquitination of RIPK1 can result in its disassociation from the TNFR1 complex where it can instead form a complex with FAS-associated death domain protein (FADD) to induce cell death either via caspase-dependent apoptosis or caspase-independent necroptosis [18]. In contrast with TNFR1, TNFR2 has a greater specificity for transmembrane bound TNFα [19], and while activation of TNFR2 can result in the canonical activation of NFκB despite the absence of a death domain, activation of TNFR2 is also associated with non-canonical activation of NFκB via activation of NFκB-inducing kinase (NIK). In cells where both receptors are expressed, predominantly immune cells, competition for the recruitment of complex forming proteins can mediate pathway cross-talk whereby an often-higher intrinsic expression of TNFR2 can negatively regulate the formation of the TNFR1 complex [20]. Thus, depending on the cellular context, TNFα can promote cell survival or cell death.

TNFα was one of the first cytokines identified as increased in brain tissue and CSF from PD patients compared with controls [21]. TNFα is also elevated in the periphery of PD patients [22] and a recent meta-analysis of over 40 subsequent different published studies using blood samples and approximately 10 using CSF confirms an increase of TNFα in PD patient biofluids [23]. It is noteworthy though that an increase is not always observed, and can depend on the analytical method employed, sample size and the ability of researchers to match groups and control for potential confounders such as the use of anti-inflammatory medications and/or other underlying inflammatory conditions.

Early post-mortem studies of PD brain tissue demonstrated the expression of TNF receptors on human dopamine neurons [24], initiating studies to determine if TNFα could directly promote neurodegeneration. Direct inoculation of TNFα into the striatum failed to induce dopaminergic neurodegeneration in rats [25]; however, a sustained lentiviral-mediated overexpression of TNFα in the substantia nigra induced extensive neurodegeneration [26]. Studies investigating the cytotoxicity of PD patient CSF on the rodent dopaminergic MES23.5 cell line failed to find a role for TNFα [27], although TNFα was toxic to primary rodent dopamine neurons in culture [28]. Further studies have indicated that the level of TNFα expression is important, with low-grade expression potentially protective, but at higher levels TNFα induces neurodegeneration [29]. Studies employing knockout mice of TNFR1 and TNFR2 have given similarly mixed results with complete neuroprotection [30] and no effect [31,32] both reported for rodents stimulated with the toxin MPTP. Knockout of TNFα itself has also been reported to be protective against MPTP [33] or have no effect on dopamine neuron degeneration induced by MPTP [34]. Such mixed results likely highlight the challenges of modelling PD, with no single model fully capturing the clinical disease course. Indeed, neutralising soluble TNFα with an engineered dominant negative variant provided neuroprotection when using either lipopolysaccharide (LPS) and 6-hydroxydopamine to induce neurodegeneration [35,36]. Support for TNFα as a therapeutic target for PD also comes from the study of inflammatory bowel disease (IBD). A study of over 10,000 patients with IBD demonstrated a 28% higher incidence of PD developing in this cohort compared with those without IBD [37]. However, the incidence of developing PD was reduced by 78% for IBD patients receiving anti-TNFα monoclonal antibody therapies, one of the many treatment options for IBD [37]. Mutations in leucine-rich repeat kinase 2 (LRRK2), the main risk factor for autosomal dominantly inherited PD, are also associated with IBD [38], and have been associated with potentiated TNFα responses [39,40] and higher levels of TNFα have been found in CSF from asymptomatic carriers of the most common LRRK2 G2019S mutation [41]. LRRK2 G2019S mice are also more susceptible to experimentally induced colitis, which was associated with increased PD pathology in the mutant mice, that could be prevented with anti-TNFα antibody therapy [42]. Thus, in general, there is support that TNFα plays an important role in PD pathophysiology; however, the complexity of the signaling pathway complicates preclinical modelling and genetic and other confounding factors may play a role in determining PD patient responses to anti- TNFα therapies.

### Interleukin 6 (IL-6)

IL-6 initiates signalling by binding to transmembrane or soluble forms of the interleukin 6 receptor (IL-6R). Biding to IL-6R triggers homodimerization of the transmembrane signalling receptor glycoprotein 130 (gp130). Binding of IL-6 to transmembrane IL-6R can activate classical IL-6/gp30 signalling in target cells expressing IL-6R (predominantly immune cells), while binding to soluble IL-6R can activate gp130 in cells that do not constitutively express IL-6R in a process known as trans IL-6 signalling [43]. Regardless of classical or trans signalling, formation of the IL-6/IL-6R/gp130 complex results in activation and phosphorylation of gp130-associated Janus kinase (JAK), which in turn facilitates recruitment, docking and phosphorylation of signal transducer and activator of transcription proteins (STAT) transcription factors. Phosphorylated STAT disassociates from the gp130 complex and forms dimers that translocate to the nucleus to regulate gene transcription [44]. IL-6-mediated gene transcription can regulate numerous biological processes including innate and acquired immunity, metabolism, development and cell survival, as well transcription of suppressor of cytokine signalling (SOCS) proteins that act as negative regulators to turn of IL-6 signalling by inhibiting phosphorylation of gp130 [45]. Activation of the JAK/STAT signalling pathway also initiates cross-talk with many other signal transduction pathways, in particular the mitogen-activated protein kinase (MAPK) and phosphoinositide 3-kinase (PI3K) pathways [44]. Thus, although generally referred to a pro-inflammatory cytokine, IL-6 is a pleiotropic cytokine with diverse cellular functions.

Like TNFα, an up-regulation of IL-6 was observed in CSF [46,47], brain tissue [48] and the periphery [49] of patients with PD over two decades ago. Large-scale meta-analysis of IL-6 in PD subsequently confirms this up-regulation across many studies [23]. Levels of IL-6 in blood have also been shown to correlate to PD severity [50], and indeed elevated levels of IL-6 may also contribute to mortality in PD [51]. Again, it should be noted though that not all studies have reported elevated IL-6 in PD patients or correlations to disease severity, and study outcomes should always be interpreted with thought given to potential inflammatory confounders. Nonetheless, overall patient results are suggestive of a chronic low-grade inflammation in PD patients and IL-6 may be an important mediator of disease.

In line with a pleiotropic role in both neuroprotection and neurodegeneration, IL-6 knockout mice actually had significantly more neurodegeneration that wild type animals following MPTP challenge [52], and this was associated with reduced activation of microglia in the IL-6 knockout animals [53]. A neuroprotective effect of low dose LPS in a neuron-astrocyte co-culture model was also dependent on IL-6, with the protective effect prevented with an IL-6 neutralizing antibody [54]. Recombinant IL-6 was also able to rescue MPTP-induced degeneration in the MN9D dopaminergic mouse cell line [55]. In contrast with a protective role, mice engineered to express high levels of IL-6 in astrocytes show early activation of microglia and age-related impaired motor function consistent with neurodegeneration [56]. When employing the α-synuclein pre-formed fibril model, IL-6 knockout mice had reduced neurodegeneration and improved motor function compared with wild-type mice with trans IL-6 signalling inducing iron accumulation in wild-type mouse neurons [57]. Moreover, in transgenic mice with the LRRK2 R1441G mutation, antibody mediated neutralisation of peripheral IL-6 was able to prevent dopamine neuron degeneration in LPS treated mice [58]. Dopamine itself can also regulate IL-6 expression in astrocytes [59]. Thus, both the models employed and levels of IL-6 attained contribute to either neuroprotective or neurodegenerative phenotypes downstream of IL-6 signalling.

### Interleukin 1 β (IL-1β)

IL-1β is a member of the IL-1 family of cytokines and shares structural similarity to IL-1α. IL-1β is produced in response to inflammatory stimuli as an inactive pro-IL-1β, which requires cleavage by caspase 1 into an active form that is secreted from the cell and able to bind the interleukin 1 receptor (IL-1R1) [60]. Activation of caspase-1 and processing of pro-IL-1β requires activation of the inflammasome complex. Inflammasomes are a family of multiprotein complexes that assemble in response to pathogenic or sterile inflammatory stimuli that serve to recruit pro-caspase-1 and initiate its self-cleavage into active caspase-1, which in turn facilitates processing of pro-IL-1β into active IL-1β (for comprehensive review see [61]). Subsequent binding of secreted IL-1β to IL-1R1 results in recruitment of the co-receptor IL-1RacP. The trimeric complex then recruits myeloid differentiation primary response 88 (MYD88) via cytoplasmic toll-interleukin-1 receptor (TIR) domains in both IL-1R1 and IL-1RacP to initiate downstream MYD88-dependent signalling resulting in activation of NFκB and induction of pro-inflammatory cytokines [62]. It is noteworthy that a second receptor, IL-1R2, can also bind IL-1β and recruit IL-1RacP. However, IL-1R2 lacks the TIR domain to initiate downstream MYD88 signalling, and thus acts as a decoy receptor to modulate the IL-1β response.

Like other proinflammatory cytokines, IL-1β is also increased in the brain and CSF of patients with PD [46,48,63]. IL-1β can also be elevated in PD patient serum [64,65], including in potential prodromal states of PD such as is plasma from asymptomatic carriers of the LRRK2 mutation [66], and CSF from patients with REM sleep behaviour disorder [67]. In particular, IL-1β is commonly used as a readout of inflammasome activation with evidence suggesting increased inflammasome induction in PD patients [68,69].

In the context of PD, early studies injecting IL-1β into rat forebrain resulted in pronounced loss of dopaminergic neurons [70]. Adenoviral mediated overexpression of IL-1β in the substantia nigra also resulted in significant neuroinflammation and dopamine neuron loss [71]. Moreover, systemic adenoviral mediated overexpression of IL-1β potentiated dopaminergic loss in the 6-hydroxydopamine rodent model of PD [72], and inhibition of IL-1R reduced the level of dopaminergic loss that was potentiated by intra nigral injection of LPS into the same model [72,73]. Consistent with a detrimental role for IL-1β, IL-1 KO mice (null for both IL-1α and IL-1β) and IL-1R1 KO mice were protected from PD pathology following intranigral [74], intraperitoneal [75] and intranasal [76] administration of LPS, respectively. Both IL-1β and IL-1R1 are also increased in rodent brain in the MPTP model of PD [77], and numerous studies have demonstrated neuroprotective effects of anti-inflammatory compounds in this PD toxin model. The role of IL-1β in PD pathogenesis may not be solely restricted to dopamine neuron loss and motor function, as lentiviral mediated overexpression of IL-1β in the hippocampus resulted in impaired cognitive function in rats [78] and elevated plasma levels of IL-1β have also been found in patients with mild cognitive impairment [79]. Important for the production of IL-1β in the context of PD, is that misfolded α-synuclein also activates the inflammasome [80,81] and pre-clinical studies indicate that inflammasome inhibition itself may be a potential therapeutic target for PD [82]. Finally, IL-1β has also been implicated in mediating toxicity of familial PD genes including *LRRK2* [83], *PINK1* [84,85] and *Parkin* [86] and it would be of interest to determine the effect of IL-1β inhibition in these genetic models to determine if this can ameliorate PD pathology.

## Key cytokines with anti-inflammatory actions that are implicated in Parkinson’s disease

Opposed to the well-known role of pro-inflammatory cytokines to induce an immune response are anti-inflammatory cytokines, which act to modulate and resolve the inflammatory response. Impaired anti-inflammatory responses can lead to potentiated or prolonged inflammation and may also contribute to PD (Figure 1).

### Interleukin 10 (IL-10)

IL-10 is the most extensively studied member of the IL-10 family of cytokines, which also includes IL-19, IL-20, IL-22, IL-24, IL-26, IL-28 and IL-29. IL-10 is secreted by most leukocyte cell types, in particular myeloid cells and T-cells, where it is produced in response to inflammatory stimuli via pattern recognition receptors or activation of T-cell receptors respectively [87]. Dimeric IL-10 initiates downstream signalling by binding to the heterodimeric IL-10Rα and IL-10Rβ receptors. Ligand binding and receptor complex formation then initiates docking and phosphorylation of JAK to IL-10Rα, and tyrosine kinase 2 (TYK2) to IL-10Rβ. Further tyrosine phosphorylation on IL-10Rα by JAK and TYK2 recruits STAT3, which is then also activated by phosphorylation and translocates to the nucleus to mediate an anti-inflammatory transcription response resulting in inhibition of NFκB-mediated pro-inflammatory cytokine production [88]. That IL-10 and IL-6 both activate the JAK-STAT pathway to mediate a predominantly anti-inflammatory and pro-inflammatory response respectively is likely due to complex cross-talk with other pathways including the MAPK and PI3K pathways to mediate the desired biological outcome. IL-10 signalling, for example, is not negatively regulated by SOCS proteins whereas IL-6 is, suggesting complex downstream regulation of JAK-STAT signalling. And again, like IL-6, IL-10 can have pleiotropic effects and can also stimulate immune responses [88].

The levels of IL-10 are increased in the serum of PD patients [49,89], a finding again supported by meta-analysis [90]. Serum levels of IL-10 also correlated to clinical measures of gastrointestinal dysfunction in patients with PD [65]. Serum [91] and plasma [92] IL-10 can also be increased in patients with Rem sleep behaviour disorder. There is less evidence for altered levels of IL-10 in PD patient CSF, but CSF levels of IL-10 were found to correlate with non-motor symptoms of depression and anxiety in PD patients [93]. Although serum levels of IL-10 are seemingly increased in PD, secretion of IL-10 was significantly impaired in PD patient PBMCs that were primed with LPS and treated with aggregated α-synuclein [94]. Knockout or kinase inhibition of LRRK2 also resulted in significantly impaired IL-10 secretion in response to infection of macrophages with mycobacterium tuberculosis [95]. Thus, the ability of IL-10 to negatively regulate inflammation may be impaired in PD.

Initial modelling studies in the context of PD demonstrated that human microglia express IL-10R and treatment of microglia cultures with IL-10 could supress LPS-mediated TNFα production [96]. Neuronal cell death induced by LPS in rat neuron-glial cultures was dependent on the activation of microglia, which was again attenuated with IL-10 treatment [97]. A similar result was also seen *in vivo*, where infusion of IL-10 could protect against LPS-mediated neurodegeneration in rodents [98]. Knockout of the IL-10R or pharmacological inhibition of JAK can prevent the protective effects of IL-10 against LPS-mediated neurodegeneration [99], further confirming the role of JAK-STAT signalling in IL-10 anti-inflammatory effects. Adenoviral mediated striatal expression of IL-10 also reduced glial activation and was neuroprotective in a 6-hydroxydopamine rat model [100] and MPTP mouse model of PD [101]. Intriguingly, IL-10 knockout mice spontaneously develop colitis [102] and have been widely used as a model for the study of inflammatory bowel disease (for review see [103]), a known risk factor for developing PD. Despite substantial evidence for a protective role of IL-10, the pleiotropic nature of IL-10 signalling is highlighted by a recent study demonstrating increased pathology and reduced survival in transgenic mice overexpressing mutant intra α-synuclein and adenoviral mediated IL-10 [104]. Thus, yet again, the models and experimental paradigms employed can result in profoundly different outcomes downstream of IL-10 signalling.

### Interleukin 4 (IL-4)

IL-4 is predominantly produced by CD4 T-cells, but can also be produced by macrophages, natural killer T-cells, innate lymphoid cells and some granulocytes. IL-4 initiates signalling by binding to one of two receptor complexes [105]. Both IL-4 receptor complexes have in common IL-4Rα. In type-1 receptor complexes, which are expressed by lymphocytes, IL-4Rα heterodimerizes with the common γ chain (γc). Type-2 receptor complexes, which are expressed by non-hemopoietic and hemopoietic non-lymphocyte cells, contain IL-4Rα and IL-13Rα1. Cells of myeloid lineage can express both type-1 and type-2 receptor complexes. Binding of IL-4 to the receptor complex results in the recruitment and activation of JAK, with type-1 receptors activating JAK1 and JAK3 and type-2 receptors activating JAK1, JAK2 and TYK2 [106]. Further tyrosine phosphorylation of IL-4Rα recruits STAT6 via its SH2 domain. STAT6 is then tyrosine phosphorylated and activated to form homodimers, resulting in translocation to the nucleus and modulation of gene expression. In lymphocytes, STAT6 activation results in differentiation of T helper 2 (T_h_2) cells via the transcription factor GATA3, and an immunoglobulin class switch in B-cells to produce IgE and some classes of IgG [107]. In macrophages, IL-4 induces a still to be understood transcriptional program that results in inhibition of NFκB activation and polarisation toward pro-repair macrophages that secrete much lower levels of pro-inflammatory cytokines. In addition to activation of JAK, insulin receptor substrate (IRS) can also bind to tyrosine phosphorylated IL-4Rα initiating activation of the downstream PI3K pathway [107].

Like other interleukin family cytokines there is some evidence for increased levels of IL-4 in CSF [46] and serum [49] of patients with PD, although meta-analysis suggests this increase is not prevalent or consistently observed [90]. Indeed, the most recent meta-analysis of inflammatory cytokines in PD suggests lower levels of IL-4 are found in PD patients [23]. However, IL-4 was also found to be increased in plasma from patients with mild cognitive impairment, and interestingly, within this cohort lower levels of IL-4 were associated with worse PD motor impairment and worse cognitive testing [108]. Longitudinal assessment of plasma IL-4 over 3 years also showed a decline in IL-4 levels over time in cognitive impaired patients [109]. This suggests that IL-4 may be increased early in PD but declines over time, potentially exacerbating a pro-inflammatory phenotype. In addition to cytokine levels, it is noteworthy that the proportion of IL-4 producing T_h_2 cells have also been found to be increased in PD patient blood [110]. The number of follicular helper T cells (Tfh) was also increased in PD patient blood and correlated to serum levels of IL-4 [111]. Indeed, a number of studies have demonstrated altered T-cell populations in PD (for review see [112]), which may be related, at least in part, to altered IL-4 signalling.

Studies evaluating the role of IL-4 in PD pathology are limited; however, recombinant IL-4 prevents LPS-induced microglial activation *in vitro* [113], and IL-4 knockout mice have enhanced microglial activation in response to LPS *in vivo* [114]. A neutralising antibody against IL-4 was also able to reduce microglial activation and ameliorate neurodegeneration in LPS treated rats [115]. Recombinant IL-4 could also prevent dopamine neuron death in murine midbrain co-cultures treated with the bioactive MPTP metabolite MPP+ [116]. In these studies, recombinant IL-4 was only protective in the presence of microglia suggesting an inhibitory effect on microglia activation and pro-inflammatory cytokine production was the principal mechanism of action. Interestingly, however, IL-4 knockout mice were not more susceptible to MPTP *in vivo* [116]. In addition, in mitochondrial toxin models of PD, the complex 1 inhibitor rotenone could prevent the protective effects of IL-4 treatment *in vitro* [117,118] suggesting therapeutic benefit of IL-4 may be limited in the pathological context of PD. Elevated levels of IL-4 are also associated with allergic inflammation via excess IgE production [105], again highlighting the pleiotropic nature and complexity of therapeutically targeting cytokine pathways.

### Interleukin 1 receptor antagonist (IL-1RA)

IL-1RA is structurally related to IL-1α and IL-1β (described above) and binds the shared IL-1R1 with similar high affinity as IL-1β. However, subtle structural differences between IL-1β and IL-1RA result in the inability of the co-receptor IL-1RacP to be recruited to the complex following binding of IL-1RA to IL-1R1 [119]. Consequently, no downstream signalling is initiated with IL-1RA acting as an inhibitory agonist of IL-1 signalling. IL-1RA also does not bind to the decoy IL-1 receptor (IL-1R2) [119]. In this manner, IL-1RA serves as an anti-inflammatory cytokine to modulate the pro-inflammatory effects of IL-1 signalling.

Less is known about the expression of IL-1RA in PD; however, lower levels of IL-1RA have been reported in PD patient serum [64] and plasma [120]. Interestingly, levels of IL-1RA correlated with the clinical assessment of fatigue in newly diagnosed PD patients [121], suggesting IL-1RA levels may also contribute to non-motor PD symptoms. In CSF, there does not appear to be evidence of altered IL-1RA levels with PD. However, it is noteworthy that in a PD cohort, levels of IL-1RA in CSF were ∼75% lower and did not correlate to levels of the same cytokine in serum [64].

Due to its anti-inflammatory properties, a number of studies have investigated the potential neuroprotective effect of IL-1RA in PD models. Anikara, a recombinant form of IL-1RA, is an approved medication for the treatment of rheumatoid arthritis. In one study, peripheral administration of Anikara was able to significantly reduce dopamine neurodegeneration in a combined LPS/6-hydroxydopamine rat model of PD, and this was associated with lower levels of pro-inflammatory cytokines [73]. Adenoviral mediated overexpression of IL-RA in the medial forebrain bundle was also able to reduce dopamine neuron loss in a combined LPS/6-hydroxydopamine rat model of PD [72]. In a rodent midbrain cell culture model, recombinant IL-1RA was able to bind to IL-1R present expressed on dopamine neurons and antagonize cell death induced by IL-1β [122]. Thus, IL-1RA may have protective effects on dopamine neurons by reducing inflammation or more directly by blocking IL-1β induced cell death. Interestingly, IL-1RA knockout mice develop motor dysfunction associated with a loss of dopamine neurons independently of any additional inflammatory stimuli [123], highlighting an important role of IL-1RA to modulate IL-1 signalling in the context of PD.

## Conclusions

In conclusion there is substantial evidence that that the immune system plays a role in PD, either by modulating the disease course and/or potentially acting as a trigger to initiate disease. As such, immunomodulatory therapies may hold promise for the treatment of PD. As outlined above however, targeting these pathways involves diverse responses due to the large levels of cross-talk and signalling pathway interplay. Moreover, the cytokines described above are only a small subset that may play a role in PD with others such as IL-12, IL-8, IL17A and interferon γ (IFNγ) also implicated (for additional reviews on cytokines in PD please see [8,124,125]). The production of cytokines is also associated with the production of chemokines, that add further diversity and complexity to inflammatory responses. As an example, the chemokine monocyte chemoattractant protein 1 (MCP1/CCL2) is important for regulating the infiltration of monocytes into brain tissue in response to α-synuclein in mice [16] and increased levels of CCL2 in CSF [126] and plasma [127] are associated with the progression/prediction of PD motor symptoms. Thus, the mechanisms by which inflammation may contribute to PD is likely vastly more complicated that outlined in this review. This also makes modelling inflammatory phenotypes in the context of PD difficult, and while many good studies have employed toxin models such as LPS and MPTP, these models do not capture the full extent of PD pathology. Such models could be complimented by evaluating immunomodulatory therapies in alpha-synuclein fibril models, or newer genetic models of PD. Despite the challenges, progress in the field is impressive and ongoing work in this complex area will continue to provide insight into the pathophysiology of PD, and hopefully offer ways to treat this debilitating disorder.

## Data Availability

Not applicable as no data were generated as part of this review.

## Abbreviations

IBD: inflammatory bowel disease
IFNγ: interferon γ
IL-10: interleukin 10
IL-1RA: interleukin 1 receptor antagonist
IRS: insulin receptor substrate
LRRK2: leucine-rich repeat kinase 2
MAPK: mitogen-activated protein kinase
MCP1: monocyte chemoattractant protein 1
MYD88: myeloid differentiation primary response 88
PD: Parkinson’s disease
PI3K: phosphoinositide 3-kinase
SOCS: suppressor of cytokine signalling
STAT: signal transducer and activator of transcription
T_h_2: T helper 2
TIR: toll-interleukin-1 receptor
TNFα: tumor necrosis factor α
TYK2: tyrosine kinase 2
